# Report of a Case of Hemorrhagic Malarial Fever

**Published:** 1890-12

**Authors:** J. R. G. Howell

**Affiliations:** Dothan, Ala.


### REPORT OF A CASE OF HEMORRHAGIC MALARIAL FEVER.

BY J. R. G. HOWELL, M. D., DOTHAN, ALA.

On the night of the 12th of October, I was called seven miles in the country to see Mr. J., aged 40 years, and of good health previous to this attack. He claimed to have been suffering for six days of severe pain in the limbs, head and lumbar region. He had been confined to his bed for three days when I first saw him, during which time the discharge from his kidneys was dark in color, in fact, he never passed urine without its looking as though it was well mixed with blood. He had epistaxis on the 11th, but none before or after during this illness. I found him at 10 o'clock P. M., of the 12th, suffering of severe frontal headache, and severe pain in the lumbar region. His temperature was 105, his circulation 120 and his respirations were 30 per minute. His skin was dry, his bowels constipated, his pupils were dilated and his tongue was covered with a dark brown coating. The tip and edges were quite red, and the entire organ seem tremulous on protrusion. I gave him five grains of antifebrin every two hours till three doses were given. At 4 o'clock A. M., of the 13th, his temperature was 102, his pulse 100, and his respiration normal. He claimed to be almost clear of pain. I took mur. tr. iron and made a saturated solution of sulph. quinine, and instructed the nurse to give him thirty drops of the solution every three hours till my return.

At 10 o'clock P. M., of the 13th, I returned to find the patient with a temperature of 104½, the circulation 120 and the respiration normal. I also found the patient vomiting almost incessantly with all the other uneasy symptoms before mentioned. I suspended the treatment used through the day and gave all the remedies in my mind for vomiting. Among others I gave sul. morphia, gr. 1-6, hypodermically, but to no good effect. Finally I applied a plaster of cantharides over the epigastric region and allowed it to remain there six hours, after which time a large blister was drawn, and when it was opened the fluid which escaped *resembled a saturated solution of permanganate of potassium.* The vomiting ceased in about four hours after the application of the plaster.

At 6 o'clock A. M., of the 14th, the temperature was 101, the pulse 100 and the patient quiet as the morning previous. At 6 o'clock this morning I began using the same solution as before, but at intervals of six, instead of three hours, using ℥*i* doses of emulsion of turpentine midway between the doses of the other medicine. I kept up this treatment each day till 9 o'clock at night, at which time I gave ⅛ gr. sul. morphia and suspended medication till morning, 6 o'clock. There was a gradual improvement from day to day, till the 20th, at which time the temperature, pulse and respiration were all normal. The urine had a natural appearance to the eye. There was, however, a little sediment on long standing. It contained no albumen or sugar and had the normal acid reaction. The patient was then instructed to take 15 drops of tr. iron after each meal. There was a rapid recovery. Now one of the questions of importance in this case to me is the color of the water in the blister before mentioned. What is the pathalogical condition of the capillaries that causes them to allow the coloring matter to escape with the liquor sanguinis?

My opinion is that the same condition is present in the kidneys which allow the coloring matter to escape with the urine, and which makes it look as though it were half blood. I would be pleased to hear from some of the *older heads* in the profession on this question.

